# A nomogram for predicting prognosis in uterine serous carcinoma: a large population-based cohort study with external validation

**DOI:** 10.3389/fonc.2026.1761938

**Published:** 2026-04-24

**Authors:** Anyang Li, Ning Xie, Jianfeng Zheng, Ruina Jiang, Siping Wang, Rongrong Zhang, Yang Sun

**Affiliations:** Department of Gynecology, Clinical Oncology School of Fujian Medical University, Fujian Cancer Hospital, Fuzhou, Fujian, China

**Keywords:** external validation, overall survival, prognostic nomogram, SEER database, uterine serous carcinoma

## Abstract

**Purpose:**

Uterine serous carcinoma (USC) is known for its aggressive behavior, high recurrence rate, and poor prognosis. Despite its clinical importance, personalized prognostic tools for USC are limited. This study aimed to develop and externally validate a nomogram to help gynecologic oncologists accurately predict patient survival and create personalized treatment regimens.

**Methods:**

A retrospective cohort study was conducted using clinical records of USC patients from the Surveillance, Epidemiology, and End Results (SEER) database (2000–2022). Patients were randomly split into training and internal validation cohorts in a 7:3 ratio. An independent external validation cohort was also used from Fujian Cancer Hospital. Prognostic factors affecting overall survival (OS) were identified using univariate and multivariate Cox regression. Model performance was evaluated using time-dependent ROC curves, calibration plots, and decision curve analysis (DCA).

**Results:**

The study included 8,204 USC patients from both SEER and Fujian Provincial Cancer Hospital cohorts. Multivariate Cox regression showed that age, FIGO stage, T stage, N stage, radiotherapy, chemotherapy, and surgery were significant independent prognostic factors for OS (all P < 0.05). The nomogram incorporating these variables displayed robust discriminatory capacity, yielding 5-year OS prediction AUC values of 0.79, 0.78, and 0.72 across the three distinct patient cohorts. Calibration plots demonstrated good agreement between predicted and observed outcomes. DCA indicated substantial clinical benefit. Survival analysis revealed significant differences in OS between the high-risk and low-risk groups (P < 0.05).

**Conclusions:**

A reliable and well−validated nomogram was established for predicting OS in USC patients. This predictive tool supports clinicians in performing individualized risk stratification, guiding patient counseling, and optimizing adjuvant therapeutic decisions.

## 1 Introduction

Endometrial cancer (EC), projected to account for approximately 69,000 new diagnoses in 2025, is the most frequently diagnosed gynecological malignancy (1). Uterine serous carcinoma (USC) constitutes a comparatively infrequent EC subtype that exhibits morphological similarities to pelvic and ovarian high-grade serous carcinomas (2, 3). In contrast to the more common endometrioid subtype (EEC), USC demonstrates limited sensitivity to hormonal influences, significant biological heterogeneity, heightened invasiveness, frequent metastatic spread, and a notably unfavorable prognosis (4, 5).

Current recommendations by the National Comprehensive Cancer Network (NCCN) guidelines advocate comprehensive surgical staging combined with supplementary adjuvant treatments, primarily involving platinum-based chemotherapy and/or radiation therapy, as the established standard care regimen for USC management (3, 6, 7). However, the therapeutic response rates to these traditional modalities are moderate at best, ranging approximately from 20% to 60%. Consequently, these limited outcomes directly contribute to persistent clinical challenges associated with USC, including frequent recurrence and heightened mortality risk (8). At initial diagnosis, over half of the patients already exhibit metastatic progression (9), and overall, the five-year survival probability is approximately half compared to that of EEC (8). Even patients diagnosed at stages I–II show a notably reduced five-year overall survival (OS) rate of 74%, compared with 86% in grade 3 EEC cases (10). These observations highlight an urgent need for improved prognostic approaches to better predict individual outcomes and inform therapeutic development for USC. Nevertheless, research on individualized prognostic modeling for USC remains scarce.

To address this clinical issue, data from the Surveillance, Epidemiology, and End Results (SEER) database were utilized to construct a nomogram predicting OS in USC patients. The performance and accuracy of the nomogram were further validated using an external single-center cohort from China. Ultimately, this study aimed to create a clinically applicable tool for gynecologic oncologists to assist with accurate prognosis evaluation and personalized management of USC patients.

## 2 Methods

### 2.1 Patient selection

**Figure 1:**
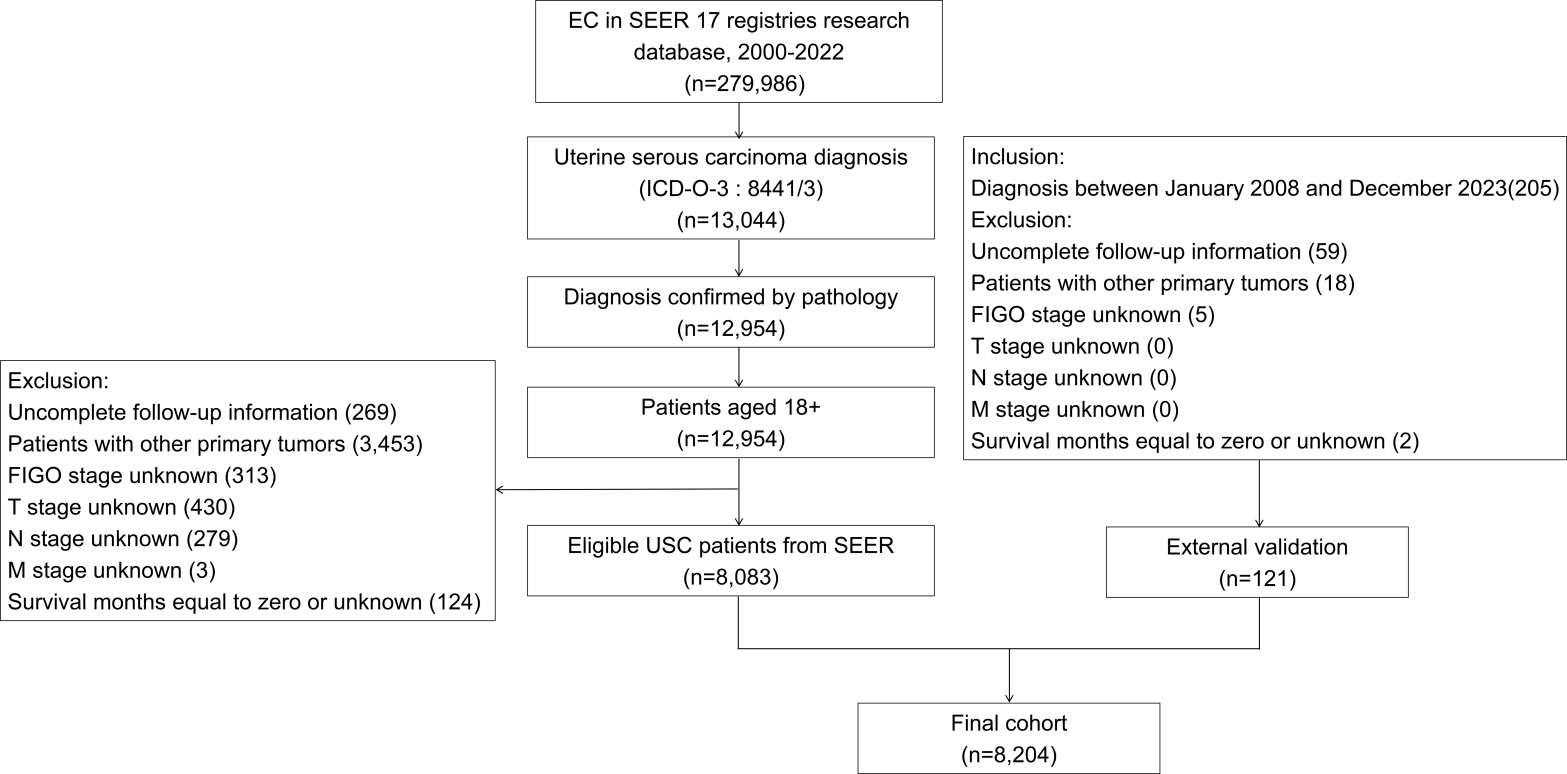
Patient selection flowchart.

A retrospective study was conducted using patient records from the SEER-17 database, identifying individuals diagnosed with pure USC between 2000 and 2022. Inclusion criteria comprised: (1) confirmed pathological diagnosis of pure USC identified through ICD-O-3 classification code 8441/3; (2) tumors primarily located at anatomical sites coded as C54.0, C54.1, C54.2, C54.3, C54.8, C54.9, or C55.9; (3) patient age of at least 18 years; and (4) detailed follow-up data availability. Excluded from this study were patients presenting with: (1) concurrent diagnosis of other primary cancers; (2) incomplete or ambiguous TNM, T, N, or M staging data; and (3) survival durations unknown or recorded as zero. Following these criteria, an independent external validation group was established, comprising pure USC patients diagnosed at Fujian Provincial Cancer Hospital from January 2008 through December 2023. The patient selection methodology is depicted in **Figure 1**.

### 2.2 Data collection

Clinicopathological characteristics were systematically gathered from the USC patients. As the study cohort included patients with both early- and advanced-stage disease, OS was selected as the primary endpoint. OS was calculated as the interval from initial pathological diagnosis to either death or the most recent follow-up. Follow-up for patients from Fujian Provincial Cancer Hospital was conducted via telephone, the study protocol was approved by the Ethics Committee of Fujian Cancer Hospital.

### 2.3 Statistics analysis

Statistical analyses were executed using R software (version 4.5.2). Differences were assessed utilizing χ² or Fisher’s exact tests. Using the time-dependent receiver operating curve (timeROC), we identified the optimal cut-off value for continuous variables associated with USC survival, including age and tumor size in order to change them into categorical variables for easily clinical application. Prognostic factors impacting OS in USC were identified through univariate and multivariate Cox regression analyses of the training cohort. The nomogram’s predictive accuracy was validated via ROC curves, and calibration plots were constructed for evaluation. Decision curve analysis (DCA) was employed to assess clinical usefulness. Internal validation was performed using the 1000 bootstrap resamples method. Risk stratification into high- and low-risk groups was performed using prognostic indices derived from the nomogram. Survival differences between groups were assessed using Kaplan–Meier analysis and log-rank tests. Additionally, hazard ratios (HRs) and corresponding 95% confidence intervals (CIs) were reported, with statistical significance threshold set at a P-value below 0.05.

## 3 Results

### 3.1 Clinicopathologic characteristics of USC patients

A total of 8,083 USC cases were enrolled, randomly separated into a training set comprising 5,658 patients and an internal validation set with 2,425 patients. A separate external validation cohort consisting of 121 cases from Fujian Cancer Hospital was also analyzed. Differences observed in clinicopathological characteristics within the external cohort might reflect regional or ethnic variations (**Table 1**).

**Table 1:** Clinicopathological characteristics of USC patients.

Characteristics	Training cohort (n = 5658)	Internal validation cohort (n = 2425)	External validation cohort (n = 121)	*P* value
Age (years)				<.001
<60	796 (14.07%)	347 (14.31%)	52 (42.98%)	
≥60	4862 (85.93%)	2078 (85.69%)	69 (57.02%)	
FIGO Stage				0.085
I	2552 (45.10%)	1059 (43.67%)	40 (33.06%)	
II	387 (6.84%)	150 (6.19%)	11 (9.09%)	
III	1505 (26.60%)	664 (27.38%)	42 (34.71%)	
IV	1214 (21.46%)	552 (22.76%)	28 (23.14%)	
T Stage				<.001
T1	3040 (53.73%)	1248 (51.46%)	55 (45.45%)	
T2	638 (11.28%)	254 (10.47%)	21 (17.36%)	
T3	1681 (29.71%)	780 (32.16%)	30 (24.79%)	
T4	299 (5.28%)	143 (5.90%)	15 (12.40%)	
N Stage				0.019
N0	4158 (73.49%)	1731 (71.38%)	75 (61.98%)	
N1	1149 (20.31%)	533 (22.98%)	33 (27.27%)	
N2	351 (6.20%)	161 (6.64%)	13 (10.74%)	
M Stage				0.570
M0	4506 (79.64%)	1906 (78.60%)	96 (79.34%)	
M1	1152 (20.36%)	519 (21.40%)	25 (20.66%)	
Grade				0.035
Grade I	99 (1.75%)	32 (1.32%)	1 (0.83%)	
Grade II	125 (2.21%)	69 (2.85%)	2 (1.65%)	
Grade III	3777 (66.76%)	1597 (65.86%)	68 (56.20%)	
Unknown	1657 (29.29%)	727 (29.98%)	50 (41.32%)	
Tumor Size (cm)				<.001
<2	1972 (34.85%)	844 (34.80%)	36 (29.75%)	
≥2	2020 (35.70%)	882 (36.37%)	65 (53.72%)	
Unknown	1666 (29.45%)	699 (28.82%)	20 (16.53%)	
Radiation				<.001
No/Unknown	3240 (57.26%)	1437 (59.26%)	51 (42.15%)	
Yes	2418 (42.74%)	988 (40.74%)	70 (57.85%)	
Chemotherapy				0.017
No/Unknown	1636 (28.91%)	714 (29.44%)	21 (17.36%)	
Yes	4022 (71.09%)	1711 (70.56%)	100 (82.64%)	
Surgery				0.007
No/Unknown	294 (5.20%)	138 (5.69%)	14 (11.57%)	
Yes	5364 (94.80%)	2287 (94.31%)	107 (88.43%)	

### 3.2 Prognostic factors of OS for USC patients

Initial univariate analyses identified patient age, FIGO classification, grade, tumor size, radiotherapy, chemotherapy, and surgical treatment as factors significantly linked to USC prognosis (P < 0.05). Further multivariate Cox regression, employing a forward stepwise approach, established age, FIGO classification, radiotherapy, chemotherapy, and surgical intervention as independent predictors influencing patient prognosis (P < 0.05) (**Table 2**). Specifically, advanced age, elevated FIGO stage, predicted poorer outcomes, whereas radiotherapy, chemotherapy, and surgical treatment were associated with improved prognosis.

**Table 2:** Univariate and multivariate cox regression analysis of OS in USC patients.

Characteristics	Univariate analysis		Multivariate analysis	
	Hazard ratio (95% CI)	*P* value	Hazard ratio (95% CI)	*P* value
Age (years)				
<60	Reference		Reference	
≥60	1.36 (1.20 ~ 1.54)	<.001	1.52 (1.34 ~ 1.72)	<.001
FIGO Stage				
I	Reference		Reference	
II	2.64 (2.22 ~ 3.13)	<.001	3.10 (2.60 ~ 3.69)	<.001
III	3.20 (2.86 ~ 3.59)	<.001	4.24 (3.76 ~ 4.79)	<.001
IV	7.19 (6.43 ~ 8.05)	<.001	8.88 (7.79 ~ 10.12)	<.001
Grade				
Grade I	Reference		Reference	
Grade II	1.19 (0.72 ~ 1.95)	0.497	0.88 (0.53 ~ 1.44)	0.604
Grade III	1.60 (1.07 ~ 2.40)	0.022	0.99 (0.66 ~ 1.49)	0.975
Unknown	1.64 (1.09 ~ 2.46)	0.018	1.01 (0.67 ~ 1.53)	0.947
Tumor Size (cm)				
<2	Reference		Reference	
≥2	1.26 (1.14 ~ 1.40)	<.001	1.06 (0.95 ~ 1.17)	0.292
Unknown	1.36 (1.23 ~ 1.51)	<.001	1.11 (1.00 ~ 1.23)	0.071
Radiation				
No/Unknown	Reference		Reference	
Yes	0.57 (0.52 ~ 0.62)	<.001	0.83 (0.75 ~ 0.92)	<.001
Chemotherapy				
No/Unknown	Reference		Reference	
Yes	0.86 (0.79 ~ 0.94)	<.001	0.47 (0.43 ~ 0.52)	<.001
Surgery				
No/Unknown	Reference		Reference	
Yes	0.17 (0.14 ~ 0.19)	<.001	0.32 (0.28 ~ 0.37)	<.001

### 3.3 Development and validation of the nomogram

**Figure 2:**
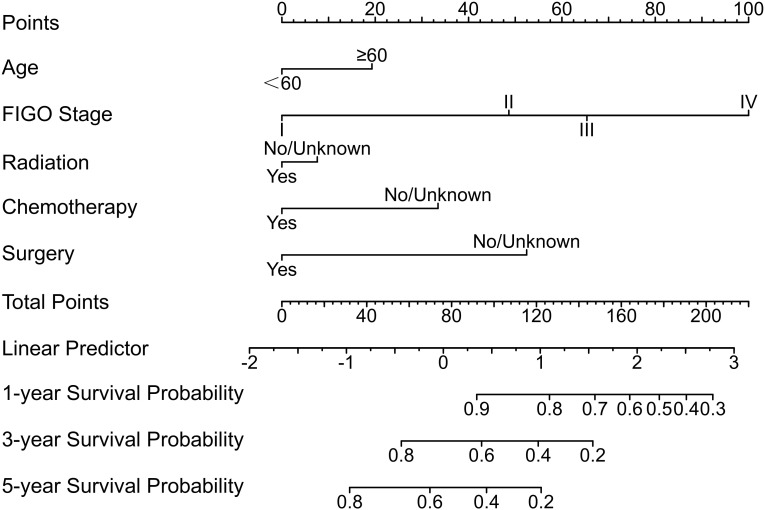
Prognostic nomogram for USC patients.

**Figure 3:**
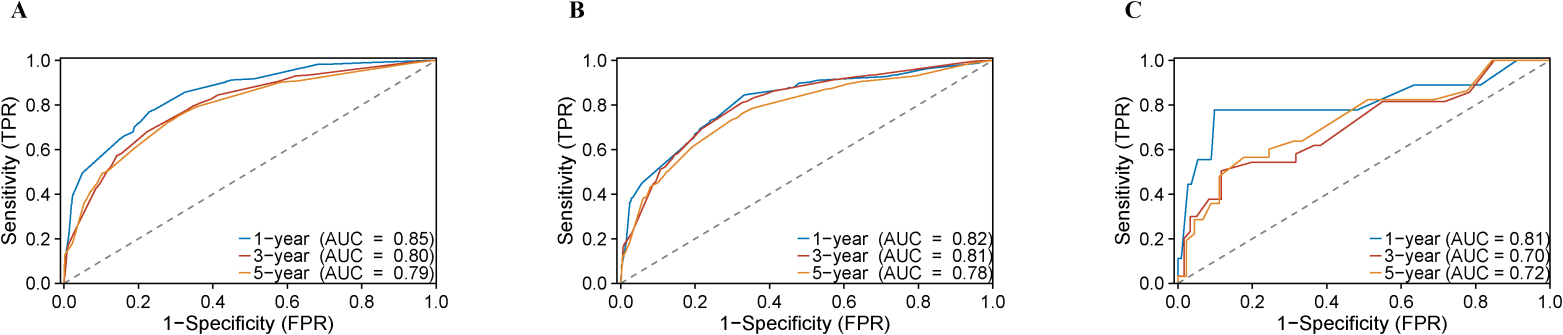
ROC curves for 1-, 3-, and 5-year OS in USC patients: **(A)** training cohort; **(B)** internal validation cohort; **(C)** external validation cohort.

**Figure 4:**
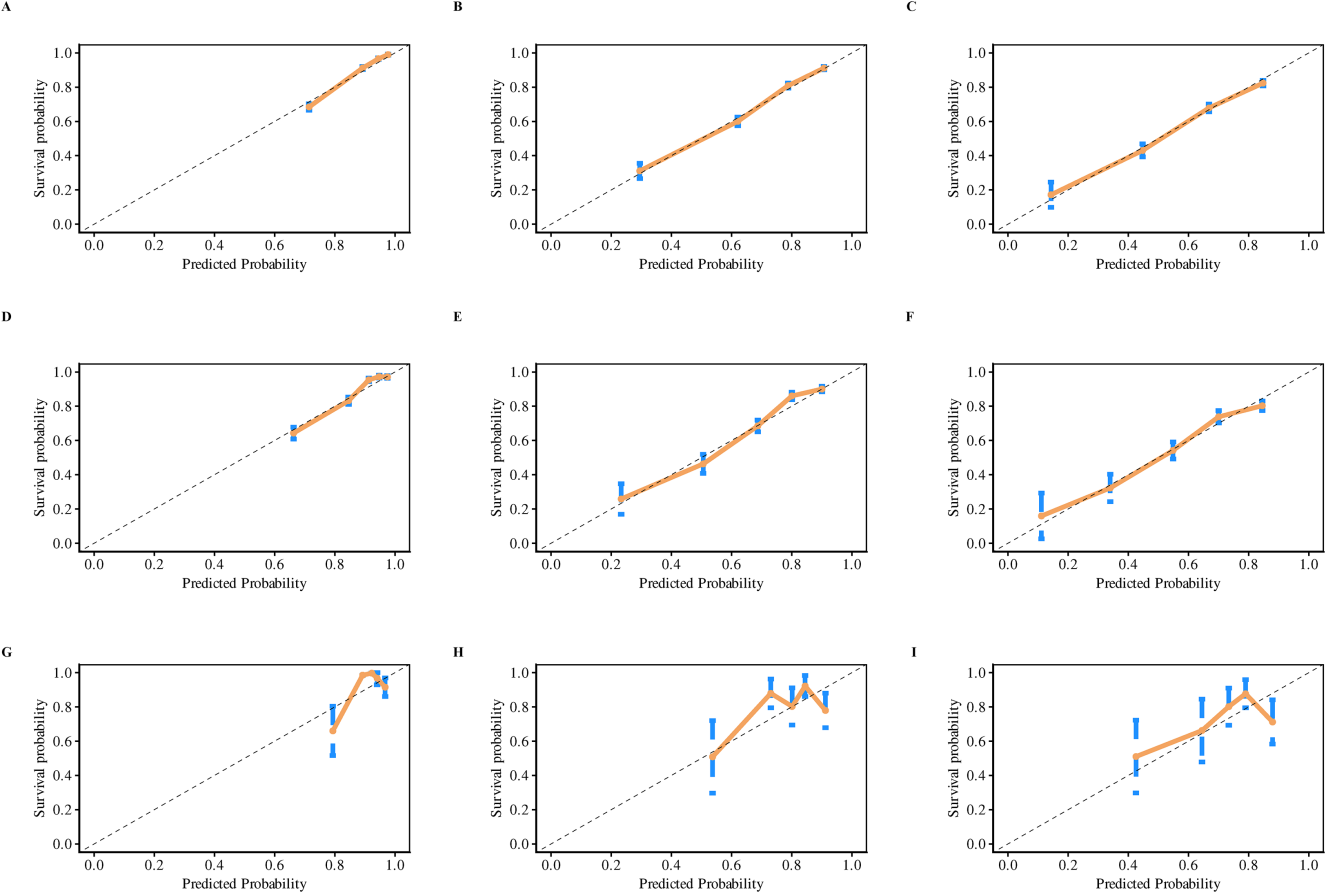
The calibration plots of the nomogram for 1-, 3-, and 5-year OS in USC patients: **(A–C)** training cohort; **(D–F)** internal validation cohort; **(G–I)** external validation cohort.

**Figure 5:**
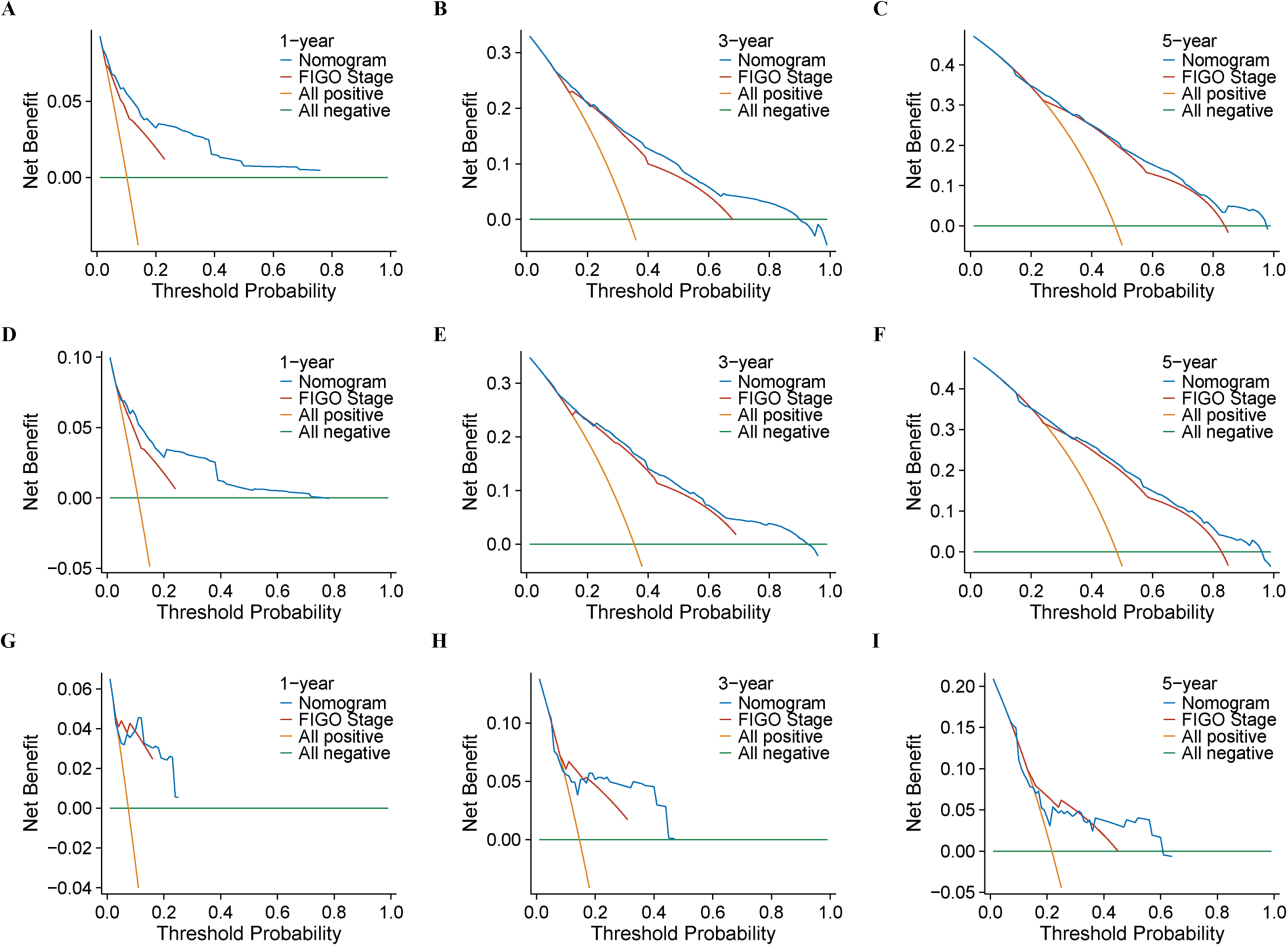
The DCA curves of the nomogram for 1-, 3-, and 5-year OS in USC patients: **(A–C)** training cohort; **(D–F)** internal validation cohort; **(G–I)** external validation cohort.

Employing results derived from multivariate Cox regression, a prognostic nomogram was developed, incorporating parameters including patient age, FIGO stage, T-stage, N-stage, radiotherapy, chemotherapy, and surgery (as depicted in **Figure 2**). This nomogram exhibited excellent predictive performance. Specifically, within the training group, the AUC values for forecasting OS at 1, 3, and 5 years were 0.85 (95% CI: 0.83–0.86), 0.80 (95% CI: 0.78–0.81), and 0.79 (95% CI: 0.77–0.81), respectively (**Figure 3A**). Similarly, in the internal validation set, AUC results for 1-, 3-, and 5-year OS predictions stood at 0.82 (95% CI: 0.79–0.85), 0.81 (95% CI: 0.79–0.83), and 0.78 (95% CI: 0.75–0.80), respectively (**Figure 3B**). Likewise, the external validation cohort demonstrated AUC values of 0.81 (95% CI: 0.63–1.00), 0.70 (95% CI: 0.57–0.87), and 0.72 (95% CI: 0.59–0.84), respectively (**Figure 3C**). Calibration plots confirmed strong consistency between observed and predicted outcomes (**Figure 4**). Additionally, decision curve analysis illustrated significant clinical utility (**Figure 5**).

### 3.4 Risk stratification

**Figure 6:**
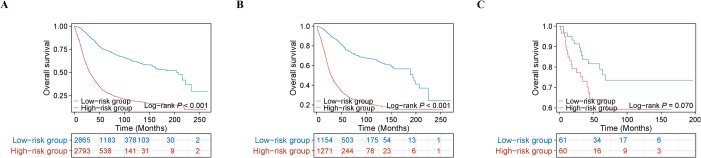
Kaplan–Meier of USC patients in different risk groups: **(A)** training cohort; **(B)** internal validation cohort; **(C)** external validation cohort.

To enhance clinical utility, a simple risk stratification scheme was devised based on the nomogram. Patients were assigned to either high-risk or low-risk categories according to the median PI value of 1.629520871 obtained from the training cohort. In all examined cohorts, Kaplan–Meier analyses paired with log-rank tests consistently indicated significantly lower OS among high-risk patients (all P < 0.05) (**Figure 6**). In the training cohort, the 5-year cumulative survival rates of the low-risk and high-risk groups were 74.9% and 30.1%, respectively. In the internal validation cohort, the 5-year cumulative survival rates of the low-risk and high-risk groups were 75.8% and 30.0%, respectively. In the external validation cohort, the 5-year cumulative survival rates of the low-risk and high-risk groups were 79.0% and 64.1%, respectively. Our constructed OS risk stratification system further verified the discriminatory ability of this prognostic nomogram.

## 4 Discussion

This study introduces the first prognostic nomogram for USC that has undergone validation outside of the SEER database. The robust predictive accuracy was confirmed through external validation using real-world patient data from China. Importantly, the prognostic variables included in the model are routinely obtainable in clinical settings, making the nomogram a potentially valuable tool for individualized risk assessment and therapeutic planning in USC.

Advanced age has long been recognized as a key determinant of prognosis in USC (11). Compared with patients aged 65–68 years, those aged 69–81 years and ≥82 years demonstrated ORs for OS of 1.50 and 1.49, respectively (12). This age−related survival disparity may be linked to TP53 alterations, which are present in more than 90% of USC cases (13). Somatic accumulation of TP53 mutations over several decades is believed to contribute to USC initiation (14), and higher mutation burdens have been associated with accelerated declines in survival among EC patients (15, 16). Evidence from the PORTEC−3 trial, which included 410 high−risk EC patients, showed a five−year recurrence−free survival of only 48% for abnormal p53 EC, substantially lower than rates observed in POLE−ultramutated (98%), MMR−deficient (72%), and NSMP (74%) subtypes (17). Moreover, diminished physiologic reserve and poorer treatment tolerance in elderly patients may further exacerbate survival outcomes.

The nomogram revealed that surgery provided the greatest survival benefit (HR = 0.32, P<0.001), confirming surgery as the cornerstone of USC treatment (18). Even in stage IV USC, surgical resection remains essential (19). However, the prognosis for USC patients receiving surgery alone remains unsatisfactory, with a survival rate of only 77% (20). Therefore, NCCN guidelines emphasize the importance of postoperative adjuvant therapy, even for patients in early-stage USC (21). A study involving 737 USC patients (FIGO stages IA–II) demonstrated that postoperative adjuvant therapy significantly improved OS compared to surgery alone (10). Adjuvant therapy also remains critically important for advanced-stage USC. Results from the PORTEC-3 trial indicated that among high-risk EC patients, stage III USC patients benefited significantly from adjuvant chemoradiotherapy (22). Similarly, a retrospective study involving 119 pure USC patients indicated that cytoreductive surgery combined with chemoradiotherapy improved survival outcomes for FIGO stage III/IV USC (23). These findings highlight the importance of surgery combined with adjuvant chemoradiotherapy, consistent with our results.

As anticipated, FIGO stage emerged as the strongest prognostic indicator for USC patients (24). Relative to FIGO stage I, patients at stage IV experienced a 4.84−fold higher mortality risk. Cherry et al. reported that FIGO staging independently predicted recurrence−free survival in uterine−confined USC, with the updated 2023 FIGO system outperforming the 2009 version in prognostic accuracy (25). Similarly, analysis of the National Cancer Database (NCDB) revealed five−year OS rates of 77%, 57%, 40%, and 17% for USC diagnosed at stages I, II, III, and IV, respectively (26).

Furthermore, the simplified risk−classification system derived from the nomogram offers practical guidance for clinicians. For individuals categorized as low−risk (PI < –0.6434), reduced intensity of postoperative adjuvant therapy and less frequent follow−up may help minimize unnecessary burden. In contrast, high−risk USC patients may benefit from escalated adjuvant treatment, including combined radiotherapy and chemotherapy, as well as emerging targeted therapies and immunotherapeutic approaches aimed at improving long−term survival (27). Lu et al. reported that, under close cardiac monitoring, the combination of carboplatin/paclitaxel with trastuzumab significantly prolonged OS compared to carboplatin/paclitaxel alone (HR = 0.56, P = 0.04) (28). In immunotherapy, the phase 3 RUBY trial demonstrated that dostarlimab combined with chemotherapy significantly improved PFS and OS among TP53abn EC patients (29).

This nomogram represents the first prognostic model for USC extending beyond the SEER database. Gynecologic oncologists should consider these findings when formulating treatment strategies for USC patients. However, this study has several limitations. First, bias was unavoidable due to its retrospective nature. For example, the SEER database does not differentiate between the ‘No’ and ‘Unknown’ for specific treatments, which may lead to classification bias. Second, the specific treatment plans, the use of targeted therapies and immune checkpoint inhibitors, may influence survival outcomes in USC patients, but such data are unavailable in the SEER database. Therefore, future studies should expand the external validation cohort and refine the content to further verify these findings in this relatively rare tumor.

## 5 Conclusions

This study is the first to construct a prognostic nomogram for USC patients based on the SEER database with external validation using a Chinese cohort. The nomogram represents a clinically feasible and practical tool that can assist gynecologic oncologists in accurately evaluating prognosis and personalizing management strategies for USC patients.

## Data Availability

The raw data supporting the conclusions of this article will be made available by the authors, without undue reservation.
